# Change in the Mortality Trend of Hospitalized Patients with Clostridium difficile Infection: A Nation-wide Study

**DOI:** 10.7759/cureus.6759

**Published:** 2020-01-23

**Authors:** Mubeen Khan Mohammed Abdul, Sanjay Bhandari

**Affiliations:** 1 Hepatology, Aurora St. Luke's Medical Center, Milwaukee, USA; 2 Internal Medicine, Medical College of Wisconsin, Milwaukee, USA

**Keywords:** clostridium difficile, mortality trends, hospitalized patients

## Abstract

Background

According to the Healthcare Cost and Utilization Project (HCUP), mortality in Clostridium difficile infection (CDI) has been rising since 2009, and an upward trend in mortality has been noted. Although there have been studies exploring the incidence of CDI and mortality in the national database, those studies were limited to one particular year. With the advent of newer modalities of diagnosis and treatment for CDI, the recent multiyear trend in disease-specific outcomes from large administrative databases is unknown.

Objective

To study the recent trend in nationwide hospital admissions and mortality along with hospital outcomes.

Methods

We queried the identified National Inpatient Sample from 2007 to 2011 to identify patients of age >18 years, with a discharge diagnosis of CDI identified by the International Classification of Diseases, 9th edition (ICD-9), clinical modification codes 008.45, respectively.

Results

We identified a decline in CDI mortality to 2.67% in 2011 as compared to 3.83% in 2007 (P<0.0001) with CDI as the primary discharge diagnosis and a downward trend in all-cause mortality from 9.2% in 2007 to 7.9% in 2011 (P<0.0001). We identified an upward trend in CDI-related hospital discharges from 2007 (N=325,022) to 2011 (N=333498). Hospital discharges with CDI as a primary discharge diagnosis also increased from 2007 (N=104,123) to 2011 (123,898). The mean length of stay decreased from 7.16 days in 2007 to 6.40 days in 2011 (P 0.0001). CDI was noted to be more common in the elderly (61-80), with a mean age of 68 years. Patients were of Caucasian descent (67%), female (64%), and primarily a Medicare payer (69%). Mean hospital charges increased from $31,551 to 35,654$ (P .04). Of interest, CDI was noted to be more common in large bed-sized non-teaching hospitals (57%) than large bed-sized teaching hospitals (42%). In terms of the geographical distribution of CDI, the southern states of the US had an increased incidence of CDI (36%) and the west coast (16%) had the least incidence.

Conclusion

Our study shows an improved trend in-hospital mortality outcomes and a decreased length of stay likely related to the advancement in CDI treatments. Hospital charges were increased from 2007 to 2011 in spite of a decrease in hospital length of stay.

## Introduction

Clostridium difficile (C. difficile) is an anaerobic, gram-positive, spore-forming bacterium that is responsible for most nosocomial diarrhea in hospitalized patients and long-term facilities [1]. The Center for Disease Control and Prevention identified Clostridium difficile infection (CDI) and upgraded the level of risk to “urgent threat" in its recent report on antibiotic resistance in the United States [2]. CDI can have variable clinical consequences, which can be mild diarrhea, pseudomembranous colitis, and toxic megacolon, and mortality can exceed 12% [3].

CDI is a major health care burden and increases hospital length of stay [4-5]. There has been a steady increase in hospital stay and hospital charges due to CDI for a decade, and as noted by the statistical brief by CDC, hospital stay has leveled off [1,6]. The data of the CDI hospitalization have not been available since 2009.

Mortality in CDI has been rising according to the Healthcare Cost and Utilization Project (HCUP; Agency for Healthcare Research and Quality, Rockville, MD) till 2009, and an upward trend in mortality has been noted [3]. Although there have been studies exploring the CDI incidence and mortality in the national database, those studies were limited to a particular year [7]. With the advent of newer modalities of treatment for CDI, the recent multiyear trend in disease-specific outcomes from large administrative databases is unknown.

## Materials and methods

Data source and objectives

Data were obtained from the Nationwide Inpatient Sample (NIS) from 2007 to 2011. We used the International Classification of Diseases, Ninth Revision Clinical Modification (ICD-9-CM) code for the diagnosis of CDI (ICD-9-CM code: 008.45). The NIS is sponsored by the Agency for Healthcare Research and Quality (AHRQ) as part of the HCUP and is the largest publicly available all-payer database in the USA. The database contains discharge-level data from about 1000 hospitals designed to approximate a 20% stratified sample of all community hospitals in the USA (1). The database contains more than 100 clinical and nonclinical elements for each hospital stay, including primary and secondary diagnoses and procedures, admission status, patient demographics, hospital characteristics, payer source, comorbidity measures, length of stay (LOS), and discharge status. Discharge weights were used to obtain national estimates.

Statistical methods

The clinical characteristics of CDI-related hospitalizations were summarized based on whether CDI was included as a primary diagnosis or all-listed diagnosis. Categorical variables were summarized with the use of respective percentages. Continuous variables, such as age, were also summarized using means with standard error (SE). We then looked into the five-year trend in CDI-related hospitalizations and other hospital outcomes like in-hospital mortality, hospital charges, and LOS. The chi-square test was used to compare categorical variables (mortality) using the Surveyfreq procedure. Similarly, the t-test was used for comparing continuous variables (hospital charges and LOS) using the Surveyfreq procedure. Appropriate survey discharge weights were applied for NIS data to obtain the national estimate. Statistical analysis was performed using SAS 9.4 software (SAS Institute Inc., Cary, NC, USA). Since NIS is a publicly available, de-identified database, it was exempt from Institutional Review Board (IRB) review.

## Results

Characteristics of CDI-related hospitalizations

**Figure 1:**
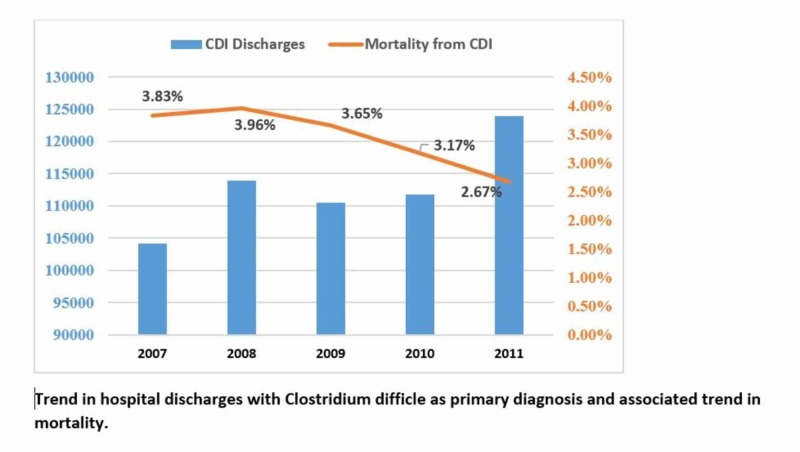
Trend in hospital discharges with Clostridium difficile as the primary diagnosis and the associated trend in mortality X-axis: years to trend; Y-axis: left, total hospital discharges; right (depicted in orange) mortality %

**Figure 2:**
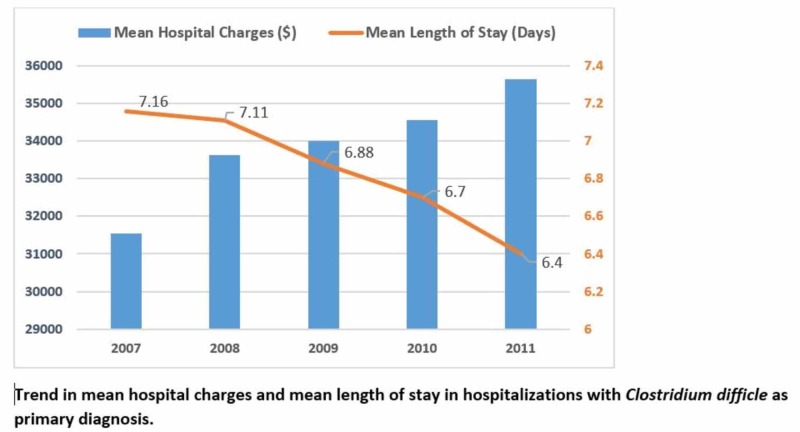
Trend in mean hospital charges and mean length of stay in hospital with Clostridium difficile as the primary diagnosis X-axis: years to trend; Y-axis: left, hospital charges, right (depicted in orange), mean length of stay

Figure 1 and Figure 2 show all the clinical characteristics of CDI-related hospitalizations. Patients with the primary diagnosis of CDI were elderly, with the age category of 61-80 years and a mean age of 68.27 ± 0.21 yrs. They were likely females (64%) and white (67%) more than black (8%) and Hispanic (6%). The majority of them had Medicare (69%) as the primary insurance and a higher burden of comorbidities (66% had Charlson Comorbidity Index >=4). In terms of geographical distribution, the Southern region (36%) had the highest incidence of CDI, whereas the Western region had the lowest incidence (16%). Interestingly, CDI tended to be more common in non-teaching (57%) and large bed-sized hospitals (62%).

Trend in CDI-related hospitalizations

When CD was included as a primary diagnosis, CDI-related hospitalizations increased by 19% from 104,123 in 2007 to 123,898 in 2011 (Table 1 and Figure 1). Similarly, when CD was included as the primary diagnosis, CDI-related hospitalizations increased by 18% from 325,022 in 2007 to 383,498 in 2011 (Table 2).

**Table 1:** Demographics of Clostridium difficile subjects

Covariates	Clostridium difficile as all Listed diagnosis	Clostridium difficile Only as a Primary Diagnosis
Age(Yrs)		
Mean	67.91(0.24)	68.27(0.21)
Median	71.8(0.22)	72.92(0.19)
Age category %		
18-40	7	9
41-60	19	19
61-80	42	40
>=81	30	32
Missing	3	3
Sex %		
Female	58	64
Male	42	36
Missing	<1	<1
Race %		
White	64	67
Black	10	8
Hispanic	7	6
Asian/Pacific Islander	2	1
Others/Missing	17	18

**Table 2:** Total hospital charges of Clostridium difficile hospitalizations CDI: Clostridium difficile infection

Outcome	2007	2008	2009	2010	2011	P-value
Total CDI discharges	104,123	113,956	110,553	111,707	123,898	
Died %	3.83	3.96	3.65	3.17	2.67	<0.0001
Hospital charge ( Mean) $						
1) All included	31,551	33,632	34,008	34,553	35,654	0.04
2) Dead excluded	30,329	32,337	32,664	33,400	34,495	0.02
Length of Stay (LOS) days						
1) All included	7.16	7.11	6.88	6.7	6.4	<0.0001
2) Dead excluded	7.04	6.7	6.77	6.6	6.32	<0.0001

Trend in CDI-related hospital outcomes

When CDI was included as a primary diagnosis, in-hospital mortality decreased from 3.83 in 2007 to 2.67 in 2011 (p<0.001) (Table 1 and Figure 1). But there was a decrease in trend in hospital charges ($31,551 in 2007 to $35,654 in 2011, p=0.04) and LOS (7.16 days in 2007 to 6.40 days in 2011, p<0.001) (Table 2 and Figure 2). A similar trend was observed when CDI was included as the all-listed diagnosis (Table 2).

## Discussion

Our study is the first one to report promising outcomes of CDI-related mortality from a large national database, trending over half a decade (2005-2011). There has been a surge in morbidity and mortality in 2004 in relation to CDI due to the development of the hypervirulent strain of North American Pulse Field referred to as NAP1/027 /BI [8]. Of note, the results are significant, especially in the era of the emergence of a hypervirulent strain of Clostridium difficile (North American Pulse Field, which is referred to as ribotype 027) [9]. Increased incidence has been reported all across the world from various international studies [10-11].

There are several interesting observations can be drawn as an explanation of the improved mortality trends in our study. First, since the identification of toxin-producing Clostridium difficile as the cause for antibiotic-associated diarrhea, several diagnostic tests were evolved over the course and the development of toxin-based assays and polymerase chain reaction facilitated the detection of both toxins A and B of Clostridium difficile [12]. It has become possible to diagnose more and more cases of CDI in hospitalized patients and, as a result, an increased number of hospital discharges were noted from 2005 to 2011 in our study. A majority of these hospital discharges related to CDI were in the elderly. Similar results were reported in a retrospective study of the US National Hospital Discharges Survey [13].

Another important observation in our study was that the number of hospital discharges remained stable from 1996-2000 with approximately 30-40 discharges per 100 thousand discharges, and it seems logical that mortality should go up with additional new cases of diagnosis, especially due to the hypervirulent strain of CDI in the latter half of 2000. Contrary to this, various treatment regimens have played a critical role in the management of CDI. Nelson et al. in their Cochrane review showed the evolution of various newer modalities of treatment in the latter half of 2000, which included fidaxomicin, vancomycin, and monoclonal antibodies [14-17].

As discussed earlier, the combination of newer and faster diagnostic modalities along with effective treatment modalities for Clostridium difficile resulted in a decrease in overall hospital length of stay, as noted in our study. The total number of hospital days were fewer as compared to the study conducted by Chen et al. [18]. Interestingly, according to a Medicare beneficiary study conducted by Drozd et al., the hospital length of stay was prolonged if CDI was identified as a secondary diagnosis [19].

The elderly population is especially at high risk for CDI. According to a retrospective study of long-term care facility data, the average age of the patients with CDI was 82, and they also identified higher three-month mortality in the elderly age group [19]. Ziakas et al., in their study, further identified the elderly population to have a high likelihood of acute care hospital admission and less likelihood of discharge to a community hospital [20]. These findings were similar to our study results. Another interesting aspect of our observations was increased CDI incidence in a nonteaching hospital. Teaching hospitals tend to have strict enforcement of isolation policies and periodic monitoring of the effectiveness of various preventive strategies to prevent outbreaks in addition to an antibiotic stewardship drive. We recommend further studies to validate our observation.

Our study has several limitations inherent in observational epidemiological studies. Our study was limited by its retrospective nature and its use of ICD-9-CM codes for the diagnosis. Many confounders that could influence the outcomes were adjusted in our study. Since our study includes only hospitalized patients, the results might be a little different from that of the outpatient setting, where the patients tend to be less severely ill. In addition, the limitation of the NIS is that isolated admissions, as opposed to isolated patients, are documented, which potentially introduces the possibility that patients with recurrent admissions may be counted as multiple events in our study.

## Conclusions

Our study shows an improved trend in hospital mortality outcomes and a decreased length of stay, likely related to advancement in CDI treatments. Hospital charges were increased from 2007 to 2011 in spite of a decrease in hospital length of stay. The period of 2007 marked the newer treatment options for CDI. Future studies are needed to evaluate the outcomes with the advent of fecal microbiota transplantation (FMT).
